# Crystal structure of (*E*)-1-(4′-methyl-[1,1′-biphen­yl]-4-yl)-3-(3-nitro­phen­yl)prop-2-en-1-one

**DOI:** 10.1107/S2056989014027443

**Published:** 2015-01-01

**Authors:** T. Vidhyasagar, K. Rajeswari, D. Shanthi, M. Kayalvizhi, G. Vasuki, A. Thiruvalluvar

**Affiliations:** a Department of Chemistry, Annamalai University, Annamalai Nagar 608 002, Tamilnadu, India; b Department of Physics, Kunthavai Naachiar Government Arts College (W) (Autonomous), Thanjavur 613 007, Tamilnadu, India; c Postgraduate Research Department of Physics, Rajah Serfoji Government College (Autonomous), Thanjavur 613 005, Tamilnadu, India

**Keywords:** crystal structure, chalcones, C—H⋯π inter­actions

## Abstract

In the title compound, C_22_H_17_NO_3_, the mol­ecule has an *E* conformation about the C=C bond, and the C—C=C—C torsion angle is −177.7 (3)°. The planes of the terminal benzene rings are twisted by 41.62 (16)°, while the biphenyl unit is non-planar, the dihedral angle between the planes of the rings being 38.02 (15)°. The dihedral angle between the nitro­phenyl ring and the inner benzene ring is 5.29 (16)°. In the crystal, mol­ecules are linked by two weak C—H⋯π inter­actions, forming rectangular tubes propagating along the *b*-axis direction.

## Related literature   

For the synthesis, anti­microbial, anti­oxidant activities and growth and characterization of π-conjugated organic non-linear optical chalcone derivatives, see: Rajendra Prasad *et al.* (2008 ▸); Lahsasni *et al.* (2014 ▸); Prabhu *et al.* (2013 ▸). For the analysis of Bovine serum albumin in the presence of some phenyl-substituted chalcones, see: Garg *et al.* (2013 ▸). For the crystal structures of related compounds, see: Shanthi *et al.* (2014 ▸); Vidhyasagar *et al.* (2015 ▸).
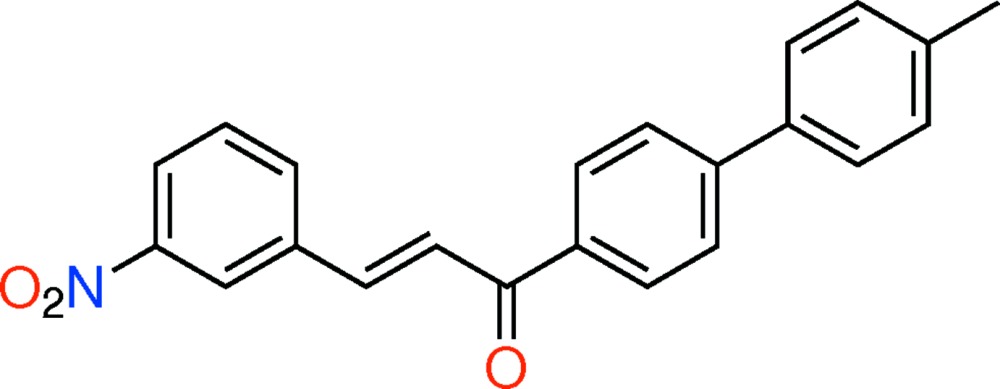


## Experimental   

### Crystal data  

C_22_H_17_NO_3_
*M* *_r_* = 343.37
Monoclinic, 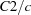

*a* = 17.8214 (10) Å
*b* = 6.1630 (3) Å
*c* = 32.3569 (19) Å
β = 103.165 (2)°
*V* = 3460.5 (3) Å^3^
*Z* = 8
Mo *K*α radiation
μ = 0.09 mm^−1^
*T* = 293 K
0.30 × 0.20 × 0.20 mm

### Data collection  

Bruker Kappa APEXII CCD diffractometer
Absorption correction: multi-scan (*SADABS*; Bruker, 2004 ▸) *T*_min_ = 0.646, *T*_max_ = 0.745
17009 measured reflections
2902 independent reflections
2058 reflections with *I* > 2σ(*I*)
*R*_int_ = 0.053

### Refinement  

*R*[*F* ^2^ > 2σ(*F* ^2^)] = 0.067
*wR*(*F* ^2^) = 0.177
*S* = 1.08
2902 reflections
236 parameters
H-atom parameters constrained
Δρ_max_ = 0.37 e Å^−3^
Δρ_min_ = −0.22 e Å^−3^

### 

Data collection: *APEX2* (Bruker, 2004 ▸); cell refinement: *APEX2* and *SAINT* (Bruker, 2004 ▸); data reduction: *SAINT* and *XPREP* (Bruker, 2004 ▸); program(s) used to solve structure: *SIR2002* (Burla *et al.*, 2003 ▸); program(s) used to refine structure: *SHELXL2014* (Sheldrick, 2008 ▸); molecular graphics: *ORTEP-3 for Windows* (Farrugia, 2012 ▸) and *PLATON* (Spek, 2009 ▸); software used to prepare material for publication: *SHELXL2014*, *PLATON* *publCIF* (Westrip, 2010 ▸).

## Supplementary Material

Crystal structure: contains datablock(s) global, I. DOI: 10.1107/S2056989014027443/su5045sup1.cif

Structure factors: contains datablock(s) I. DOI: 10.1107/S2056989014027443/su5045Isup2.hkl

Supporting information file. DOI: 10.1107/S2056989014027443/su5045Isup3.cdx

Supporting information file. DOI: 10.1107/S2056989014027443/su5045Isup4.cml

**Fig. 1.**
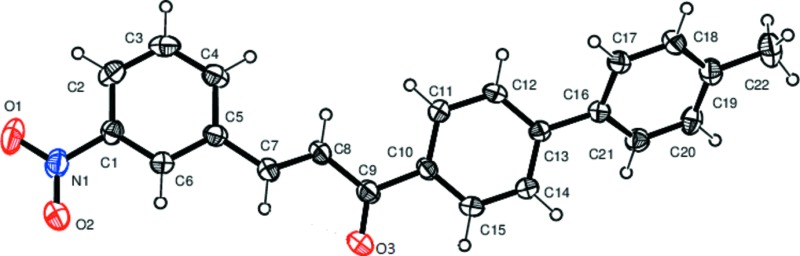
The molecular structure of the title compound, with atom labelling. Displacement ellipsoids are drawn at the 30% probability level.

. DOI: 10.1107/S2056989014027443/su5045fig1.tif
The mol­ecular structure of the title compound, with atom labelling. Displacement ellipsoids are drawn at the 30% probability level.

**Fig. 2.**
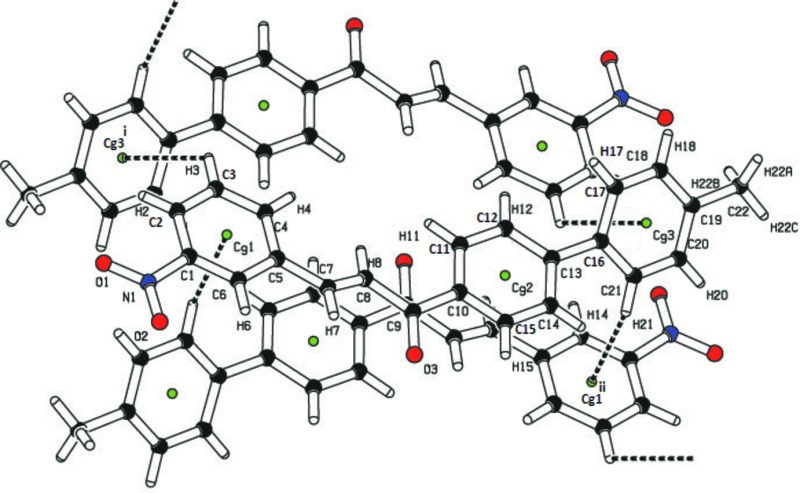
The partial packing of the title compound, showing the two weak C—H···π interactions (see Table 1 for details).

. DOI: 10.1107/S2056989014027443/su5045fig2.tif
The partial packing of the title compound, showing the two weak C—H⋯π inter­actions (see Table 1 for details).

CCDC reference: 1039539

Additional supporting information:  crystallographic information; 3D view; checkCIF report

## Figures and Tables

**Table 1:** Hydrogen-bond geometry (, ) *Cg*1 and *Cg*3 are the centroids of rings C1C6 and C16C21, respectively.

*D*H*A*	*D*H	H*A*	*D* *A*	*D*H*A*
C3H3*Cg*3^i^	0.93	2.99	3.531(4)	119
C21H21*Cg*1^ii^	0.93	2.94	3.607(3)	129

Symmetry codes: (i) 
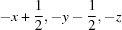
; (ii) 
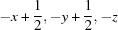
.

## supplementary crystallographic information

### S1. Comment

Synthesis and antimicrobial activity of some chalcones derivatives have been reported (Rajendra Prasad *et al.*, 2008). The synthesis, characterization and evaluation of antioxidant activities of some novel chalcone analogues have been reported (Lahsasni *et al.*, 2014). The analysis of Bovine serum albumin in the presence of some phenyl substituted chalcones have been reported (Garg *et al.*, 2013). The growth and characterization of π conjugated organic non-linear optical chalcone derivatives were reported (Prabhu *et al.*, 2013). The crystal structures of related compounds were reported (Shanthi *et al.*, 2014; Vidhyasagar *et al.*, 2015). As part of our on-going research on biphenyl chalcone derivatives, the title compound, was synthesized and its crystal structure is reported on herein.

In the title compound, Fig. 1, the molecule exists as an E conformer with the C5—C7—C8—C9 torsion angle being -177.7 (3)°. In the molecule, the terminal benzene rings (C1—C6 and C16—C21) are twisted by an angle of 41.62 (16)°, while the biphenyl part (C10—C15 and C16—C21) is non-planar, the dihedral angle between the rings being 38.02 (15)°. The dihedral angle between the nitrophenyl ring (C1—C6) and the inner phenyl ring (C10—C15) is 5.29 (16)°.

In the crystal, there are two weak C3—H3···π and C21—H21···π interactions (Table 1 and Fig. 2) involving the terminal methylbenzene ring (C16—C21) and the terminal nitrobenzene ring (C1—C6), respectively. This results in the formation of rectangular tubes propagating along [010]. No classic hydrogen bonds are observed.

### S2. Experimental

A mixture of 4-acetyl-4'-methylbiphenyl (3.43 g, 10 mmol) and 3-nitro benzaldehyde (1.07 g, 10 mmol) in ethanol (25 ml) in the presence of NaOH (10 ml 30%) were heated in a water bath for 30 min. and then allowed to cool. The solid that separated was filtered and recrystallized from ethanol. The yellow crystals of the title compound, used for the X-ray diffraction study, were grown by slow evaporation of a solution in acetone (yield: 2.5 g, 70%).

### S3. Refinement

All H-atoms were positioned geometrically and allowed to ride on their parent atoms, with C—H = 0.93 - 0.96 Å with *U*_iso_(H) = 1.5*U*_eq_(C) for methyl H atoms and = 1.2*U*_eq_(C) for other H atoms.

### Crystal data

**Table:**

C_22_H_17_NO_3_	*F*(000) = 1440
*M**_r_* = 343.37	*D*_x_ = 1.318 Mg m^−^^3^
Monoclinic, *C*2/*c*	Melting point: 462.3 K
Hall symbol: -C 2yc	Mo *K*α radiation, λ = 0.71073 Å
*a* = 17.8214 (10) Å	Cell parameters from 5055 reflections
*b* = 6.1630 (3) Å	θ = 2.4–23.5°
*c* = 32.3569 (19) Å	µ = 0.09 mm^−^^1^
β = 103.165 (2)°	*T* = 293 K
*V* = 3460.5 (3) Å^3^	Block, yellow
*Z* = 8	0.30 × 0.20 × 0.20 mm

### Data collection

**Table:**

Bruker Kappa APEXII CCD diffractometer	2902 independent reflections
Radiation source: fine-focus sealed tube	2058 reflections with *I* > 2σ(*I*)
Graphite monochromator	*R*_int_ = 0.053
ω and φ scan	θ_max_ = 24.7°, θ_min_ = 2.4°
Absorption correction: multi-scan (*SADABS*; Bruker, 2004)	*h* = −20→20
*T*_min_ = 0.646, *T*_max_ = 0.745	*k* = −7→7
17009 measured reflections	*l* = −37→37

### Refinement

**Table:**

Refinement on *F*^2^	0 restraints
Least-squares matrix: full	Hydrogen site location: inferred from neighbouring sites
*R*[*F*^2^ > 2σ(*F*^2^)] = 0.067	H-atom parameters constrained
*wR*(*F*^2^) = 0.177	*w* = 1/[σ^2^(*F*_o_^2^) + (0.0622*P*)^2^ + 6.9426*P*] where *P* = (*F*_o_^2^ + 2*F*_c_^2^)/3
*S* = 1.08	(Δ/σ)_max_ < 0.001
2902 reflections	Δρ_max_ = 0.37 e Å^−^^3^
236 parameters	Δρ_min_ = −0.22 e Å^−^^3^

### Special details

**Table:**

Geometry. Bond distances, angles *etc*. have been calculated using the rounded fractional coordinates. All su's are estimated from the variances of the (full) variance-covariance matrix. The cell e.s.d.'s are taken into account in the estimation of distances, angles and torsion angles

### Fractional atomic coordinates and isotropic or equivalent isotropic displacement parameters (Å^2^)

**Table:**

	*x*	*y*	*z*	*U*_iso_*/*U*_eq_	
O1	0.5576 (2)	−0.0419 (5)	−0.18992 (10)	0.1050 (16)	
O2	0.57708 (17)	0.2525 (5)	−0.15451 (8)	0.0826 (11)	
O3	0.39470 (16)	0.5129 (4)	0.01333 (8)	0.0742 (10)	
N1	0.55103 (17)	0.0708 (6)	−0.16008 (9)	0.0619 (11)	
C1	0.50961 (16)	−0.0197 (5)	−0.12946 (9)	0.0448 (10)	
C2	0.48178 (19)	−0.2286 (6)	−0.13485 (11)	0.0571 (12)	
C3	0.4412 (2)	−0.3050 (5)	−0.10630 (12)	0.0619 (14)	
C4	0.42788 (19)	−0.1752 (5)	−0.07404 (11)	0.0543 (12)	
C5	0.45527 (18)	0.0367 (5)	−0.06918 (9)	0.0442 (10)	
C6	0.49828 (17)	0.1111 (5)	−0.09729 (9)	0.0427 (10)	
C7	0.43736 (19)	0.1877 (5)	−0.03729 (9)	0.0517 (11)	
C8	0.38766 (19)	0.1586 (5)	−0.01312 (10)	0.0524 (11)	
C9	0.37335 (18)	0.3262 (5)	0.01629 (10)	0.0495 (11)	
C10	0.33265 (17)	0.2675 (5)	0.05025 (9)	0.0411 (10)	
C11	0.30439 (18)	0.0609 (5)	0.05492 (10)	0.0481 (11)	
C12	0.26955 (18)	0.0165 (5)	0.08822 (10)	0.0479 (11)	
C13	0.26254 (17)	0.1744 (5)	0.11789 (9)	0.0398 (9)	
C14	0.2909 (2)	0.3785 (5)	0.11259 (10)	0.0527 (11)	
C15	0.32487 (19)	0.4245 (5)	0.07947 (10)	0.0525 (11)	
C16	0.22680 (17)	0.1252 (5)	0.15388 (9)	0.0411 (10)	
C17	0.23877 (18)	−0.0738 (5)	0.17499 (10)	0.0484 (11)	
C18	0.20358 (19)	−0.1232 (5)	0.20751 (10)	0.0527 (11)	
C19	0.15524 (19)	0.0243 (6)	0.22083 (10)	0.0529 (11)	
C20	0.14488 (18)	0.2233 (6)	0.20100 (10)	0.0542 (11)	
C21	0.17998 (18)	0.2742 (5)	0.16818 (10)	0.0491 (11)	
C22	0.1162 (2)	−0.0328 (8)	0.25609 (12)	0.0813 (18)	
H2	0.49001	−0.31504	−0.15695	0.0685*	
H3	0.42262	−0.44648	−0.10885	0.0742*	
H4	0.40014	−0.23004	−0.05526	0.0652*	
H6	0.51930	0.24991	−0.09416	0.0512*	
H7	0.46418	0.31838	−0.03384	0.0616*	
H8	0.36098	0.02805	−0.01481	0.0628*	
H11	0.30885	−0.04782	0.03569	0.0576*	
H12	0.25041	−0.12199	0.09077	0.0572*	
H14	0.28687	0.48747	0.13186	0.0631*	
H15	0.34298	0.56398	0.07669	0.0626*	
H17	0.27127	−0.17533	0.16688	0.0578*	
H18	0.21237	−0.25769	0.22078	0.0632*	
H20	0.11360	0.32586	0.20984	0.0650*	
H21	0.17207	0.41032	0.15553	0.0588*	
H22A	0.15172	−0.10892	0.27806	0.1225*	
H22B	0.07234	−0.12346	0.24514	0.1225*	
H22C	0.09953	0.09766	0.26755	0.1225*	

### Atomic displacement parameters (Å^2^)

**Table:**

	*U*^11^	*U*^22^	*U*^33^	*U*^12^	*U*^13^	*U*^23^
O1	0.131 (3)	0.115 (3)	0.090 (2)	−0.020 (2)	0.069 (2)	−0.045 (2)
O2	0.102 (2)	0.080 (2)	0.0823 (19)	−0.0289 (17)	0.0551 (17)	−0.0140 (16)
O3	0.104 (2)	0.0562 (16)	0.0751 (17)	−0.0230 (15)	0.0470 (16)	−0.0017 (13)
N1	0.0566 (18)	0.072 (2)	0.063 (2)	−0.0032 (16)	0.0260 (15)	−0.0152 (17)
C1	0.0358 (16)	0.051 (2)	0.0489 (18)	0.0039 (15)	0.0121 (14)	−0.0019 (15)
C2	0.052 (2)	0.051 (2)	0.067 (2)	0.0034 (17)	0.0106 (17)	−0.0158 (18)
C3	0.062 (2)	0.0386 (19)	0.083 (3)	−0.0083 (17)	0.012 (2)	−0.0014 (18)
C4	0.056 (2)	0.049 (2)	0.058 (2)	−0.0107 (16)	0.0130 (16)	0.0048 (16)
C5	0.0493 (18)	0.0413 (18)	0.0405 (16)	−0.0064 (15)	0.0069 (14)	0.0018 (14)
C6	0.0444 (17)	0.0391 (17)	0.0449 (17)	−0.0019 (14)	0.0109 (14)	−0.0005 (14)
C7	0.064 (2)	0.050 (2)	0.0460 (18)	−0.0110 (16)	0.0227 (16)	−0.0018 (15)
C8	0.060 (2)	0.053 (2)	0.0498 (19)	−0.0167 (17)	0.0244 (17)	−0.0032 (16)
C9	0.053 (2)	0.052 (2)	0.0444 (18)	−0.0119 (16)	0.0133 (15)	−0.0002 (15)
C10	0.0451 (17)	0.0422 (18)	0.0381 (16)	−0.0045 (14)	0.0139 (13)	0.0057 (14)
C11	0.058 (2)	0.0442 (18)	0.0455 (18)	−0.0050 (16)	0.0189 (16)	−0.0059 (14)
C12	0.057 (2)	0.0345 (17)	0.056 (2)	−0.0096 (15)	0.0207 (16)	0.0027 (14)
C13	0.0437 (17)	0.0346 (16)	0.0430 (16)	0.0010 (13)	0.0136 (14)	0.0000 (13)
C14	0.074 (2)	0.0369 (18)	0.0525 (19)	−0.0066 (17)	0.0257 (18)	−0.0051 (15)
C15	0.069 (2)	0.0367 (18)	0.055 (2)	−0.0110 (16)	0.0211 (17)	−0.0010 (15)
C16	0.0449 (18)	0.0383 (17)	0.0405 (16)	−0.0025 (14)	0.0109 (14)	−0.0005 (13)
C17	0.055 (2)	0.0412 (18)	0.0533 (19)	0.0002 (15)	0.0214 (16)	0.0005 (15)
C18	0.063 (2)	0.050 (2)	0.0464 (19)	−0.0043 (17)	0.0155 (17)	0.0082 (15)
C19	0.051 (2)	0.064 (2)	0.0443 (18)	−0.0056 (17)	0.0122 (15)	−0.0019 (17)
C20	0.052 (2)	0.065 (2)	0.0488 (19)	0.0109 (17)	0.0180 (16)	−0.0065 (17)
C21	0.0530 (19)	0.0433 (19)	0.0508 (18)	0.0059 (16)	0.0117 (16)	0.0006 (15)
C22	0.084 (3)	0.110 (4)	0.059 (2)	−0.005 (3)	0.035 (2)	0.002 (2)

### Geometric parameters (Å, º)

**Table:**

O1—N1	1.217 (4)	C17—C18	1.376 (5)
O2—N1	1.210 (5)	C18—C19	1.387 (5)
O3—C9	1.223 (4)	C19—C20	1.377 (5)
N1—C1	1.473 (4)	C19—C22	1.507 (5)
C1—C2	1.376 (5)	C20—C21	1.386 (5)
C1—C6	1.367 (4)	C2—H2	0.9300
C2—C3	1.379 (5)	C3—H3	0.9300
C3—C4	1.378 (5)	C4—H4	0.9300
C4—C5	1.391 (4)	C6—H6	0.9300
C5—C6	1.394 (4)	C7—H7	0.9300
C5—C7	1.477 (4)	C8—H8	0.9300
C7—C8	1.321 (5)	C11—H11	0.9300
C8—C9	1.466 (4)	C12—H12	0.9300
C9—C10	1.493 (4)	C14—H14	0.9300
C10—C11	1.390 (4)	C15—H15	0.9300
C10—C15	1.382 (4)	C17—H17	0.9300
C11—C12	1.388 (5)	C18—H18	0.9300
C12—C13	1.392 (4)	C20—H20	0.9300
C13—C14	1.381 (4)	C21—H21	0.9300
C13—C16	1.480 (4)	C22—H22A	0.9600
C14—C15	1.375 (5)	C22—H22B	0.9600
C16—C17	1.396 (4)	C22—H22C	0.9600
C16—C21	1.389 (4)		
			
O1—N1—O2	122.9 (3)	C19—C20—C21	121.5 (3)
O1—N1—C1	118.1 (3)	C16—C21—C20	121.1 (3)
O2—N1—C1	119.0 (3)	C1—C2—H2	121.00
N1—C1—C2	119.4 (3)	C3—C2—H2	121.00
N1—C1—C6	118.2 (3)	C2—C3—H3	119.00
C2—C1—C6	122.4 (3)	C4—C3—H3	119.00
C1—C2—C3	117.6 (3)	C3—C4—H4	120.00
C2—C3—C4	121.1 (3)	C5—C4—H4	120.00
C3—C4—C5	121.0 (3)	C1—C6—H6	120.00
C4—C5—C6	117.8 (3)	C5—C6—H6	120.00
C4—C5—C7	123.0 (3)	C5—C7—H7	116.00
C6—C5—C7	119.1 (3)	C8—C7—H7	116.00
C1—C6—C5	120.1 (3)	C7—C8—H8	119.00
C5—C7—C8	127.5 (3)	C9—C8—H8	119.00
C7—C8—C9	121.9 (3)	C10—C11—H11	120.00
O3—C9—C8	120.6 (3)	C12—C11—H11	120.00
O3—C9—C10	119.9 (3)	C11—C12—H12	119.00
C8—C9—C10	119.5 (3)	C13—C12—H12	119.00
C9—C10—C11	123.4 (3)	C13—C14—H14	119.00
C9—C10—C15	118.4 (3)	C15—C14—H14	119.00
C11—C10—C15	118.2 (3)	C10—C15—H15	119.00
C10—C11—C12	120.2 (3)	C14—C15—H15	119.00
C11—C12—C13	121.6 (3)	C16—C17—H17	119.00
C12—C13—C14	117.3 (3)	C18—C17—H17	119.00
C12—C13—C16	121.4 (3)	C17—C18—H18	119.00
C14—C13—C16	121.3 (3)	C19—C18—H18	119.00
C13—C14—C15	121.6 (3)	C19—C20—H20	119.00
C10—C15—C14	121.3 (3)	C21—C20—H20	119.00
C13—C16—C17	121.3 (3)	C16—C21—H21	119.00
C13—C16—C21	121.7 (3)	C20—C21—H21	119.00
C17—C16—C21	117.0 (3)	C19—C22—H22A	110.00
C16—C17—C18	121.5 (3)	C19—C22—H22B	110.00
C17—C18—C19	121.1 (3)	C19—C22—H22C	109.00
C18—C19—C20	117.8 (3)	H22A—C22—H22B	109.00
C18—C19—C22	120.6 (3)	H22A—C22—H22C	109.00
C20—C19—C22	121.7 (3)	H22B—C22—H22C	109.00
			
O1—N1—C1—C2	1.7 (5)	C15—C10—C11—C12	0.0 (5)
O1—N1—C1—C6	−176.3 (3)	C9—C10—C15—C14	−177.2 (3)
O2—N1—C1—C2	−178.1 (3)	C11—C10—C15—C14	0.7 (5)
O2—N1—C1—C6	3.9 (5)	C10—C11—C12—C13	−0.7 (5)
N1—C1—C2—C3	−178.0 (3)	C11—C12—C13—C14	0.8 (5)
C6—C1—C2—C3	0.0 (5)	C11—C12—C13—C16	−178.7 (3)
N1—C1—C6—C5	176.0 (3)	C12—C13—C14—C15	−0.1 (5)
C2—C1—C6—C5	−1.9 (5)	C16—C13—C14—C15	179.3 (3)
C1—C2—C3—C4	1.2 (5)	C12—C13—C16—C17	37.9 (4)
C2—C3—C4—C5	−0.3 (5)	C12—C13—C16—C21	−142.1 (3)
C3—C4—C5—C6	−1.7 (5)	C14—C13—C16—C17	−141.5 (3)
C3—C4—C5—C7	175.0 (3)	C14—C13—C16—C21	38.5 (5)
C4—C5—C6—C1	2.7 (5)	C13—C14—C15—C10	−0.6 (5)
C7—C5—C6—C1	−174.1 (3)	C13—C16—C17—C18	−177.7 (3)
C4—C5—C7—C8	−8.8 (5)	C21—C16—C17—C18	2.3 (5)
C6—C5—C7—C8	167.9 (3)	C13—C16—C21—C20	177.9 (3)
C5—C7—C8—C9	−177.7 (3)	C17—C16—C21—C20	−2.1 (5)
C7—C8—C9—O3	15.0 (5)	C16—C17—C18—C19	−0.6 (5)
C7—C8—C9—C10	−164.7 (3)	C17—C18—C19—C20	−1.3 (5)
O3—C9—C10—C11	177.9 (3)	C17—C18—C19—C22	179.2 (3)
O3—C9—C10—C15	−4.4 (5)	C18—C19—C20—C21	1.4 (5)
C8—C9—C10—C11	−2.4 (5)	C22—C19—C20—C21	−179.1 (3)
C8—C9—C10—C15	175.3 (3)	C19—C20—C21—C16	0.3 (5)
C9—C10—C11—C12	177.7 (3)		

### Hydrogen-bond geometry (Å, º)

Cg1 and Cg3 are the centroids of rings C1–C6 and C16–C21, respectively.

**Table:**

*D*—H···*A*	*D*—H	H···*A*	*D*···*A*	*D*—H···*A*
C3—H3···*Cg*3^i^	0.93	2.99	3.531 (4)	119
C21—H21···*Cg*1^ii^	0.93	2.94	3.607 (3)	129

Symmetry codes: (i) −*x*+1/2, −*y*−1/2, −*z*; (ii) −*x*+1/2, −*y*+1/2, −*z*.
