# Supplementary figures and images for: Crystal structure of (E)-1-(4′-methyl-[1,1′-biphen­yl]-4-yl)-3-(3-nitro­phen­yl)prop-2-en-1-one
Source: Acta Crystallogr E Crystallogr Commun. 2015 Jan 1;71(Pt 1):o65–6. doi: 10.1107/S2056989014027443 (PMC4331888; doi:10.1107/S2056989014027443)

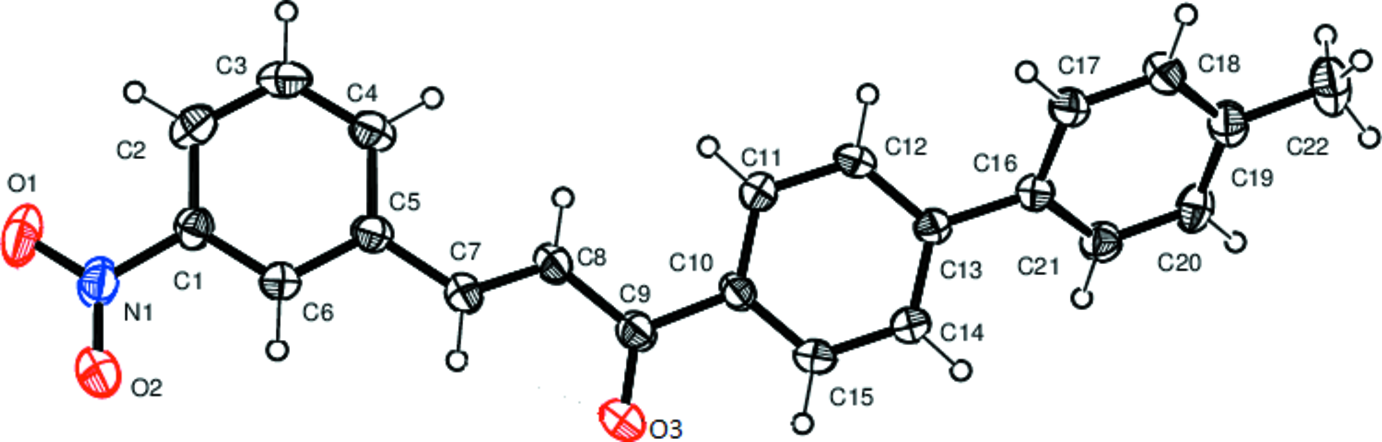

Supplement: Supplementary file 5 [file e-71-00o65-fig1.tif]

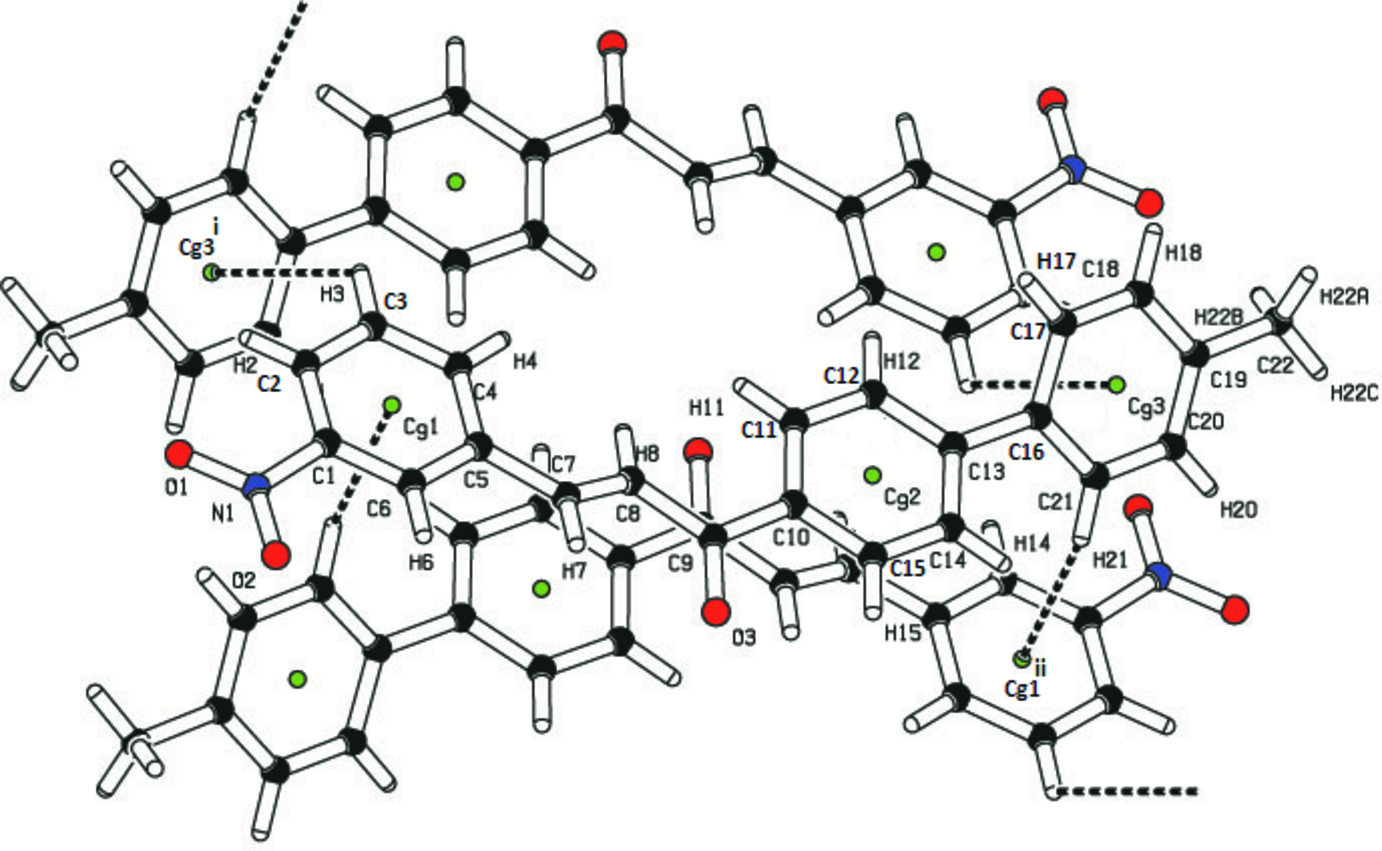

Supplement: Supplementary file 6 [file e-71-00o65-fig2.tif]
